# Antimicrobial and antioxidant activity of encapsulated tea polyphenols in chitosan/alginate-coated zein nanoparticles: a possible supplement against fish pathogens in aquaculture

**DOI:** 10.1007/s11356-024-32058-x

**Published:** 2024-01-23

**Authors:** Dmitri Fabrikov, Ágnes Timea Varga, María Carmen Vargas García, Péter Bélteky, Gábor Kozma, Zoltán Kónya, Josefa L. López Martínez, Fernando Barroso, María José Sánchez-Muros

**Affiliations:** 1 Department of Biology and Geology, University of Almería-CEIMAR Marine Campus of International Excellence, Almería, Spain; 2 Department of Applied and Environmental Chemistry, University of Szeged, Szeged, Hungary; 3 MTA, Reaction Kinetics and Surface Chemistry Research Group, Rerrich Béla tér 1, Szeged, H-6720 Hungary; 4 Department of Organic Chemistry, University of Almería, Almería, Spain

**Keywords:** EGCG, Green tea extract, Biopolymer, Marine pathogens, Nanomaterial, Layer-by-layer, Antibiotic

## Abstract

Regulation of antibiotic use in aquaculture calls for the emergence of more sustainable alternative treatments. Tea polyphenols (GTE), particularly epigallocatechin gallate (EGCG), have various biological activities. However, tea polyphenols are susceptible to degradation. In this work, EGCG and GTE were encapsulated in zein nanoparticles (ZNP) stabilized with alginate (ALG) and chitosan (CS) to reduce the degradation effect. ALG-coated ZNP and ALG/CS-coated ZNP encapsulating EGCG or GTE were obtained with a hydrodynamic size of less than 300 nm, an absolute *ζ*-potential value >30 mV, and an encapsulation efficiency greater than 75%. The antioxidant capacity of the encapsulated substances, although lower than that of the free ones, maintained high levels. On the other hand, the evaluation of antimicrobial activity showed greater efficiency in terms of growth inhibition for ALG/CS-ZNP formulations, with average overall values of around 60%, reaching an inhibition of more than 90% for *Photobacterium damselae*. These results support encapsulation as a good strategy for tea polyphenols, as it allows maintaining significant levels of antioxidant activity and increasing the potential for antimicrobial activity, in addition to increasing protection against sources of degradation.

**Supplementary Information:**

The online version contains supplementary material available at 10.1007/s11356-024-32058-x.

## Introduction

Aquaculture reached in 2020 46% of the total fish production industry. Since the1990s, aquaculture has grown 300%, reaching a production of 87.5 million tons (FAO 2022). However, this fast growth of the aquaculture industry brought several challenges, such as the elimination of pathogens (Pérez-Sanchez et al. 2018), feed utilization (Encarnação 2016), contamination (Hai et al. 2020), and sustainability (Boyd et al. 2020).

Infectious diseases in aquatic animals cause a loss of 6 billion US dollars per year (Stentiford et al. 2017); this amounts to 50% of the aquaculture industry located in developing countries (Assefa and Abunna 2018). Diseases affect growth performance, increasing mortality and lowering the marketability of affected animals, which is exacerbated by saturated and stressful environments such as aquaculture productions (Lafferty et al. 2015). Bacterial infections in aquatic animals are mainly caused by Gram-negative bacteria such as *Aeromonas* spp., *Vibrio* spp., and *Pseudomonas* spp. and Gram-positive bacteria *Streptococcus* spp. and *Staphylococcus* spp. (Preena et al. 2020). Infectious diseases not only affect aquaculture production but increase the spread of pathogens to wild animals, causing an impact in the capture industry and the aquatic ecosystem (Diana 2009).

Antibiotics have been one of the main chemicals used in aquaculture to combat infectious diseases, although in recent years their use has decreased due to the increase in antibiotic-resistant bacteria and the regulation of their use (Regulation (EU) 2019/6), thus limiting the number of antibiotics allowed and the cases where they can be applied (Lulijwa et al. 2020). New strategies to combat or prevent infections have emerged in recent years, such as vaccines (Mondal and Thomas 2022) and feed supplemented immunostimulants (Wang, Sun, Liu, and Xue 2017), nanomaterials (Okeke et al. 2022).

Polyphenols are secondary metabolites produced as a response to stress by plants. More than 10,000 polyphenols can be found in nature and can be classified by their structure as flavonoids (catechins, quercetin, curcumin) and non-flavonoids (gallic acid, cinnamic acid, resveratrol). Polyphenols have a wide range of biological activities, and several studies showed their antibacterial, antioxidant, growth promoter, anti-inflammatory, immunostimulant, and antihyperglycemic activities in aquatic animals (Ahmadi et al. 2022; Bouarab-Chibane et al. 2019; Imperatore et al. 2023; Taguri et al. 2006; Tinh et al. 2021; Yang et al. 2021; Yuan et al. 2021).

Tea polyphenols are mainly composed of flavonoids, where catechins represent to 80-90% of total flavonoids in tea leaves (*Camellia sinensis*). Epigallocatechin gallate (EGCG) is the main catechin in tea leaves, representing 50-80% of total catechins and the most biologically active compound among other polyphenols found in tea (Kim et al. 2014; Singh et al. 2011; Yan et al. 2020). Inclusion of tea catechins in aquaculture showed positive results in growth performance, antibacterial, immunostimulant, antiviral, and antioxidant activities, among others (Ji et al. 2018; Li et al. 2022; Qian et al. 2021; Thawonsuwan et al. 2010; Wang, Sun, and Zhu 2017; Zhang et al. 2020; Zhang et al. 2021). However, tea polyphenols have disadvantages, as they exhibit low stability in biological media; they are easily degraded by physiologically relevant temperature, oxygen concentration, and metal ion content at alkaline and neutral pH as well (Jin et al. 2022a; Krupkova et al. 2016; Xu et al. 2019). Therefore, oral administration of tea polyphenols leads to low absorption and a short half-life (Dang et al. 2013). To overcome these disadvantages, nanoencapsulation has become a strategy to deliver these labile molecules (Dang et al. 2015; Di Santo et al. 2021; Rambaran 2020).

Nanotechnology has different applications in aquaculture, such as the delivery of nutraceutical molecules and vaccines, water purification, pathogen detection, antimicrobial and antiviral activity, and preservation of products (Fajardo et al. 2022; Shah and Mraz 2020). Biopolymer nanocarriers have gained relevance for the delivery of active compounds due to their low toxicity and biodegradability (Faridi Esfanjani and Jafari 2016). Zein is a hydrophobic protein found in corn; its low water solubility allows the formation of colloidal nanoparticles in water (Pascoli et al. 2018) and has been widely used for the encapsulation of hydrophilic and hydrophobic active compounds (Jin et al. 2022b; Nunes et al. 2020; Zhang et al. 2014; Zheng et al. 2022). However, dispersions of zein nanoparticles exhibit low colloidal stability, a tendency toward aggregation, and precipitation at pH 5–7 (Yuan et al. 2022). Coating zein nanoparticles with biopolymers improves their stability and the presence of different functional groups facilitates the interaction between nanoparticles and bioactive molecules, thus increasing encapsulation (Yuan et al. 2022). Among the biopolymers with the ability to improve the stability of zein nanoparticles, two polymers that occur naturally in the marine environment stand out: alginate and chitosan (Carrasco-Sandoval et al. 2021; Jiang et al. 2021; Khan et al. 2019; Loureiro et al. 2022; Pauluk et al. 2019; Wu et al. 2023). These polymers are low in toxicity, biodegradable, and biocompatible (Abdel-Ghany and Salem 2020; Lee and Mooney, 2012). Alginate and chitosan have been used separately or together for the elaboration of encapsulation matrices for the delivery of bioactive substances in aquaculture (Masoomi Dezfooli et al. 2019), growth promoters and immunostimulants (Abdel-Ghany and Salem 2020; Neamat-Allah et al. 2019; van Doan et al. 2016; Yudiati et al. 2019).

In this study, we have carried out the encapsulation of green tea extract and EGCG in zein nanoparticles stabilized by a layer-by-layer technique with alginate and chitosan. These nanomaterials were characterized and evaluated for their potential to develop activities of interest in aquaculture, such as antioxidant capacity compared to free compounds in solution, and antimicrobial activity against Gram-negative and Gram-positive pathogens of importance in aquaculture.

## Materials and methods

### Compounds and reactants

Corn zein, low-viscosity sodium alginate, and 1,1-diphenyl-2-picrylhydrazyl free radical (DPPH^·^) were obtained from Tokyo Chemical Industries (Tokyo, Japan). Chitosan of low molecular weight (135 kDa) was obtained from Sigma-Aldrich (St. Louis, MO, USA). Epigallocatechin gallate 98% (EGCG) was purchased from Biosynth (Staad, Switzerland). Green tea extract (GTE) with a content of 44% EGCG was obtained from Herbadirekt (Wetzlar, Germany). Potassium persulfate 99% and 2,2′-azino-bis-(3-ethylbenzthiazoline-6-sulfonic acid) diammonium salt 98% (ABTS^·^) were purchased from Glentham Life Science (Corsham, UK). Glacial acetic acid, absolute ethanol, methanol, and culture medium were obtained from Labbox (Barcelona, Spain).

### Nanoparticle synthesis

**Fig. 1:**
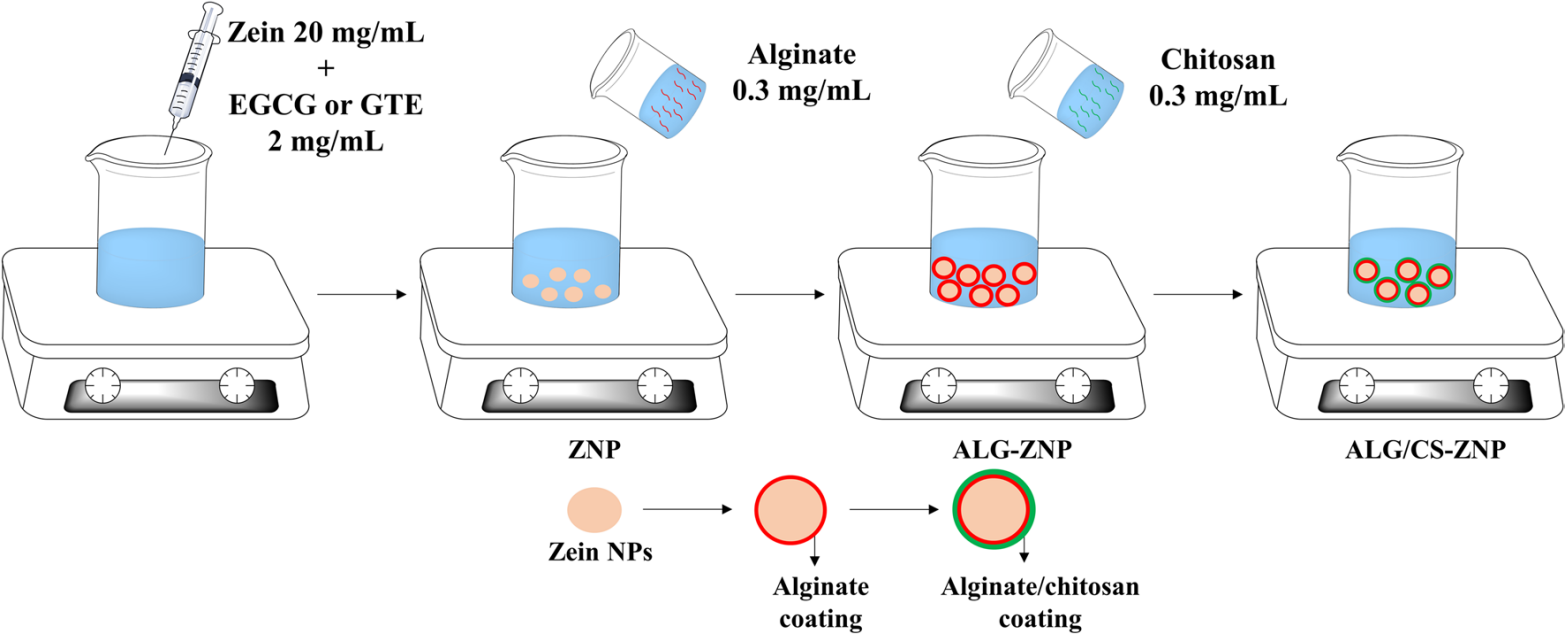
Experimental procedure for zein nanoparticle synthesis and coating with alginate and chitosan

Zein nanoparticle (ZNP) preparation has been carried out using the antisolvent precipitation method (Fig. 1) based on previous studies with some modifications (Jin et al. 2022b; Khan et al. 2019). Briefly, ZNPs were prepared by adding 4 mL of ethanolic (80:20) zein solution (20 mg/mL) into 16 mL of distilled water and stirred magnetically for 30 min. Ethanol was removed by rotatory evaporator at 35 °C for 20 min and the loss of volume was compensated with distilled water.

The coating process was carried out by applying the layer-by-layer method based on electrostatic deposition. The ZNP dispersion was added to an alginate solution of the same volume prepared at different concentrations (0.2, 0.3, 0.4, and 0.5 mg/mL) and stirred magnetically for 30 min. Alginate (ALG)-coated zein nanoparticles (ALG-ZNP) were mixed with the same volume of a chitosan (CS) acetic acid solution (1%) at different concentrations (0.2, 0.3, and 0.4 mg/mL) and stirred magnetically for 30 min therefore obtaining alginate/chitosan-coated ZNPs (ALG/CS-ZNP). Polyphenol loading was carried out by adding EGCG or GTE (2 mg/mL) to the starting ethanolic solution of zein, leading to ALG-ZNP-E and ALG/CS-ZNP-E or ALG-ZNP-T and ALG/CS-ZNP-T particles, respectively, depending on whether the synthesis was concluded after the ALG coating step or the formation of the CS shell.

### Characterization of nanoparticles

#### Encapsulation efficiency

Encapsulation efficiency (EE) was obtained based on the method described by Pantoja-Vale et al. (2022). The nanoparticle dispersions were centrifuged at 18,000 *g* for 20 min at 4 °C (Orto Alresa Biocen 22r, Spain). The polyphenol content of the supernatant was quantified with spectrophotometer (Power Wavex, USA) at 274 nm for both EGCG and green tea extract by applying their calibration curves, *y*=12.013*x* + 0.1275, *R*^2^=0.9989 and *y*=9.246*x*+0.0903, *R*^2^=0.9999 respectively. The encapsulation efficiency was calculated using the following equation:

1 $$\textrm{EE}\%=\left(1-\frac{C_{\textrm{Sup}}}{C_{\textrm{R}}}\right)\ast 100$$

where *C*_Sup_ is the concentration of EGCG or GTE in the supernatant and *C*_R_ is the theoretical concentration of EGCG or GTE.

#### Hydrodynamic size, polydispersity index, and ζ-potential

Average hydrodynamic diameter, polydispersity index (PDI), and *ζ*-potential of nanoparticles were evaluated using a Zetasizer-Nano ZS instrument (Malvern Instruments, Malvern, UK). Nanoparticle dispersions were diluted (1:10) prior to analysis and measured in a disposable folded capillary zeta cell (Malvern Instruments, Malvern, UK).

#### Scanning electron microscopy (SEM)

SEM images were obtained with a HITACHI S-3500N instrument with an acceleration voltage of 3 kV. The samples were coated with gold prior to measurement.

#### X-ray diffraction (XRD)

Nanoparticle dispersions were lyophilized with a Telstar Lyoquest-55 instrument for 48 h at −50 °C. XRD measurements between 5 and 80 (2θ°) were performed with a D8 Advance Diffractometer (Bruker, Germany) at a current of 50 kV and 50 mA.

#### Fourier transform infrared (FTIR) spectroscopy

FTIR measurements were performed with a Bruker Vertex 70 instrument (32 scans, 4 cm^−1^ resolution) with the KBr pellet technique and compressed to tablets. The transmittance of the 4000–400 cm^−1^ wavenumber region was recorded.

### Antibacterial assay

The lyophiles of *Vibrio anguillarum* (CECT 522), *Vibrio alginolyticus* (CECT 436), *Photobacterium damselae* (CECT 5122), *Pseudomonas anguilliseptica* (CECT 899), and *Streptococcus iniae* (CECT 7363) were obtained from the Spanish Type Culture Collection of the University of Valencia (Valencia, Spain). Lyophiles were recovered and maintained following the instruction provided by CECT. The inocula of all bacteria were grown in TSB 1% NaCl at 37 °C for *S. iniae*, 30 °C for *V. anguillarum* and *V. alginolyticus*, and 25 °C for *P. anguilliseptica* and *P. damselae*. The concentration of each inoculum was adjusted to 0.5 OD at 600 nm. The assay was prepared by adding 0.5 mL of standardized inoculum and 0.5 mL of freshly prepared nanoparticle dispersion into 4 mL of TSB 1% NaCl in a test tube. Oxytetracycline (10 μg/mL) was used as a positive control. Free EGCG and GTE (1 mg/mL) were also tested. Blank tubes were prepared adding 0.5 mL of nanoparticle dispersions into 4.5 mL of culture medium. Three tubes were prepared for each sample.

After 48 h of incubation in a thermostatized agitated water bath, sample absorbance was recorded at 600 nm. The calculation of inhibitory rate (IR%) was conducted following the next equation:

2 $$\textrm{IR}\ \left(\%\right)=\frac{\left(I-{I}_B\right)-\left({A}_S-{A}_{BS}\right)}{\left(I-{I}_B\right)}\ast 100$$

where *A*_*S*_ is the absorbance of the inoculum with nanoparticle dispersion or active compound, *A*_*BS*_ is the absorbance of its respective blank, *I* is the absorbance of the inoculum, and *I*_*B*_ is the absorbance of the culture medium.

### Antioxidant activity

To examine the antioxidant activity of prepared nanoparticles, DPPH and ABTS tests have been carried out following the methods described by Xiao et al. (2020). The data obtained from this assay, as well as those corresponding to the antimicrobial activity, can be consulted in the repository of the University of Almeria, through the link http://hdl.handle.net/10835/14882.

### Statistical analyses

The analysis was performed in triplicate using the ANOVA one-way test; the results are expressed as mean ± SE. Subsequently, a means comparison (Tukey’s HSD test) was carried out; the significance level was established as *P*<0.05. All calculations were performed using IBM SPSS Statistics 28 software (2022).

## Results and discussion

### Characterization of nanoparticles

#### Particle size, polydispersity index, and ζ-potential

Nanoparticle dispersions have been characterized by their hydrodynamic size, polydispersity index, and *ζ*-potential. Table 1 shows the effects on ZNP when they were coated with alginate at different concentrations. ZNP with no coating were smaller than ALG-ZNP at any concentration of alginate used.

**Table 1:** Size, polydispersity index, and *ζ*-potential of zein nanoparticles coated with different alginate concentrations (ALG-ZNP).

ALG (mg/mL)	Hydrodynamic size (nm)	PDI	ZP (mV)
0	113.90 ± 1.37^a^	0.161 ± 0.003	+20.63 ± 0.86^a^
0.2	138.80 ± 1.47^bc^	0.172 ± 0.013^a^	−11.27 ± 0.40^b^
0.3	142.06 ± 1.22^cd^	0.182 ± 0.014^ab^	−33.63 ± 0.83^c^
0.4	145.97 ± 1.95^b^	0.195 ± 0.004^a^	−42.57 ± 0.83^d^
0.5	152.80 ± 3.05^d^	0.234 ± 0.004^bc^	−53.90 ± 0.44^e^

Means (± SE) of three individual measurements. The same letter within a row is not significantly different from each other (*P* < 0.05)

*PDI* polydispersity index, *ZP ζ*-potential

The hydrodynamic size of ALG-ZNP increased as the concentration of ALG in solution increased. Similar results were obtained for alginate/zein nanoparticles by Carrasco-Sandoval et al. (2021). A possible explanation is that the size increased as more layers of ALG were deposited on the surface of the nanoparticle (Jiang et al. 2021). PDI shows a trend similar to that shown for particle size, that is, increased ALG concentration increased PDI, which means a reduction in homogeneity of the dispersion (Raval et al. 2019).

The *ζ*-potential of ZNP changed from positive to negative when negatively charged ALG was introduced into the system, thus stabilizing the dispersion of ZNP by increasing its absolute charge value at higher concentrations. After the addition of 0.2 mg/mL ALG, the measured *ζ*-potential was −11.27 mV and precipitation was observed after some minutes of stirring. This may be due to the neutralization of the highly positive charge of zein and the negative charge of alginate, resulting in a low stability dispersion at this concentration (Khan et al. 2019). After evaluating these results, the ALG concentration of 0.3 mg/mL was selected as it gave a smaller particle size and PDI, while *ζ*-potential absolute potential value was >30 mV, which indicates that the dispersion was electrostatically stable (Samimi et al. 2019).

Table 2 shows the hydrodynamic size, PDI, and *ζ*-potential of ALG-ZNP coated with chitosan at different concentrations. The size was higher for the ALG/CS-ZNP dispersions than for the ALG-ZNP.

**Table 2:** Hydrodynamic size, polydispersity index (PDI), and *ζ*-potential (ZP) of zein/alginate nanoparticles coated with different concentrations of chitosan (ALG/CS-ZNP)

CS (mg/mL)	Hydrodynamic size (nm)	PDI	ZP (mV)
0.2	212.60 ± 4.71^b^	0.196 ± 0.005^a^	42.10 ± 2.16^a^
0.3	199.60 ± 2.55^a^	0.198 ± 0.011^a^	40.27 ± 1.39^a^
0.4	271.83 ± 1.07^c^	0.303 ± 0.024^b^	52.10 ± 1.18^b^

Means (± SE) of three individual measurements. The same letter within a row is not significantly different from each other (*P* < 0.05)

*PDI* polydispersity index, *ZP ζ*-potential

No significant differences were observed for PDI and *ζ*-potential between 0.2 and 0.3 mg/mL chitosan solution. When the concentration of the chitosan solution reached 0.4 mg/mL, the PDI and *ζ*-potential increased significantly due to the excess of chitosan in the dispersion. Due to these results, the formulation used for the encapsulation of EGCG and GTE was 0.3 mg/mL for the alginate and chitosan solutions.

Regarding the encapsulation efficiency of EGCG and GTE of ZNP, levels of 45% and 58% were achieved, respectively (data not shown).

After coating with ALG, an increase could be observed (Table 3), reaching 75% and 80% of encapsulation, respectively. Upon the addition of chitosan to the system, encapsulation increased to 82% and 85%, respectively.

**Table 3:** Hydrodynamic size, polydispersity index (PDI), *ζ*-potential (ZP), and encapsulation efficiency (EE) of different formulations

Formulation	Hydrodynamic size (nm)	PDI	ZP (mV)	EE %
ALG-ZNP-E	143.73 ± 0.68^a^	0.245 ± 0.005^b^	−31.47 ± 0.12^a^	75.24 ± 0.97^a^
ALG-ZNP-T	144.03 ± 0.18^a^	0.202 ± 0.002^a^	−30.73 ± 0.49^a^	80.13 ± 1.13^b^
ALG/CS-ZNP-E	238.90 ± 0.65^b^	0.297 ± 0.001^d^	42.93 ± 0.28^b^	82.04 ± 1.15^b^
ALG/CS-ZNP-T	266.47 ± 0.66^c^	0.270 ± 0.007^c^	41.97 ± 0.38^b^	85.14 ± 0.49^c^

Means (± SE) of three individual measurements. The same letter within a row is not significantly different from each other (*P* < 0.05)

*PDI* polydispersity index, *ZP ζ*-potential, *EE* encapsulation efficiency

These results are similar to those obtained by other studies (Jin et al. 2022b; Liang et al. 2021) based on the encapsulation of EGCG in ZNP, although with different stabilization compounds. The DLS analysis (Table 3) of EGCG and GTE encapsulated in ZNP coated with ALG and CS displayed higher PDI values for all formulations, although the hydrodynamic size increased for ALG/CS-ZNP-E and ALG/CS-ZNP-T compared to blank nanoparticles. The *ζ*-potential did not vary substantially and, therefore, it can be concluded that the addition of EGCG or GTE did not alter the colloidal properties of the particles.

#### Scanning electron microscopy (SEM)

**Fig. 2:**
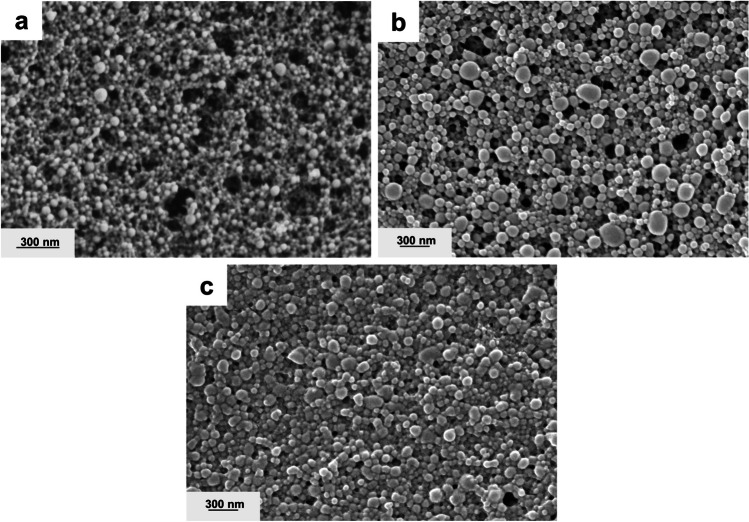
Scanning electron microscope images of ZNP (**a**), ALG-ZNP (**b**), and ALG/CS-ZNP

Figure 2 shows SEM pictures of the three nanoparticle structures prepared throughout our experiments.

The image of ZNP (Fig. 2a) displays spherical structures with smooth surfaces, although a small adhesion of the particles can be observed. This may be due to the film-forming capacity of zein during solvent evaporation (Jiang et al. 2021). The particles had an average size of 30 nm, which is significantly smaller than the size observed in the DLS measurements. The difference may be mainly due to the dehydration and consequent shrinkage of the particles. In addition, particles within aggregates can be visually distinguished, whereas DLS measurements cannot distinguish between well-dispersed single particles and compact aggregates. The ALG-ZNP image (Fig. 2b) also displayed spherically shaped particles, but the size was larger than that of ZNP. The mean particle size was 92 nm, although bigger particles were also visible. The addition of CS layer to nanoparticles did not change the size observed by SEM regarding ALG-ZNP (Fig. 2). However, increased adhesion can be observed between particles, as well as a layer in which the particles are embedded. These results are similar to those obtained in studies analyzing the same materials (Khan et al. 2019; Khan et al. 2021; Pauluk et al. 2019). Lin et al. (2020) obtained similar results for carboxymethyl chitosan-coated zein nanoparticles. The film-like structure could improve the dispersibility of the nanoparticles after the drying process and the release rate of the encapsulated compound.

#### X-ray diffraction (XRD) analyses

**Fig. 3:**
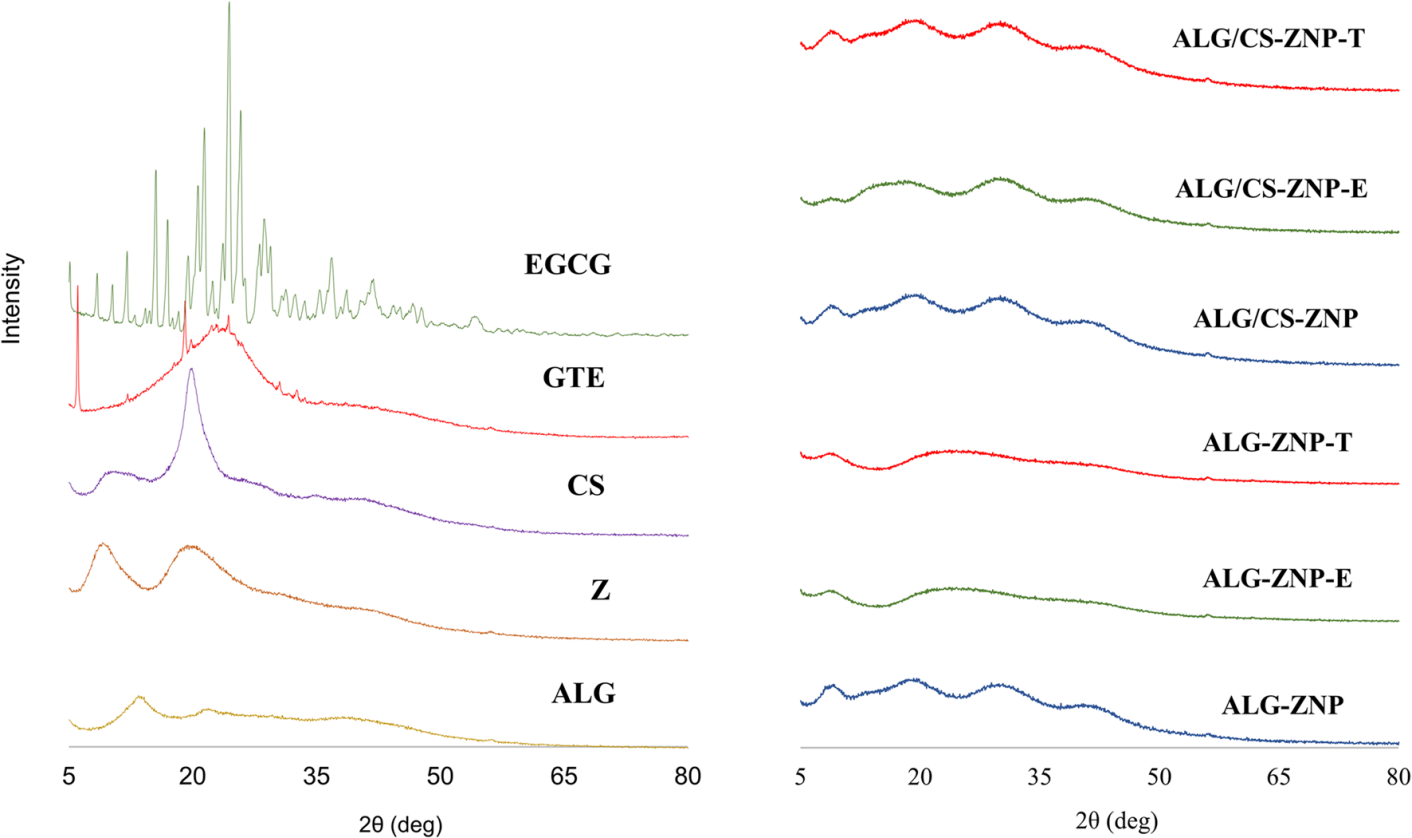
X-ray diffractograms of starting materials and nanoparticle powders

The X-ray diffractogram of the starting materials and nanoparticle powders can be observed in Fig. 3, where zein shows two wide characteristic absorption peaks at 9.5° and 20° related to the α-helix structure within zein (Jiang et al. 2021). The sodium alginate diffractogram showed two broad peaks at 13.4° and 21.8°, while chitosan showed two peaks at 10° and 20°. This showed that both polysaccharides had semicrystalline characteristics, which is consistent with previous studies (Bhagyaraj et al. 2020; Ju et al. 2020; Sundarrajan et al. 2012). EGCG has characteristic sharp peaks at 15.5°, 17°, 20.5°, 21.4°, 24.4°, and 25.8° and several small sharp peaks up to 50°. This demonstrated the crystalline properties of EGCG (Fang et al. 2019; Xie et al. 2021; Zhao et al. 2022a). The green tea extract showed a broad peak from 10 to 35°, and some sharp peaks can be observed at 6°, 19°, and 24.3°.

The X-ray diffractograms of ALG/CS-ZNP-E and ALG/CS-ZNP-T did not show the characteristic sharp peaks present in EGCG or green tea extract, indicating that the encapsulated compounds did not exist in crystalline form and that EGCG and green tea extract may be encapsulated within the nanoparticles (Gao et al. 2021; Jin et al. 2022a). It can also be seen that the characteristic peaks of the polymers decreased in intensity, probably due to interactions between the polymers themselves and the encapsulated compounds (Jin et al. 2022b; Khan et al. 2019). Supplementary data (Figure S1) show the physical mixture of both ALG/CS-ZNP-E and ALG/CS-ZNP-T. In the physical mixture containing EGCG, some of the sharp peaks denoted crystalline polyphenol can be seen with a decrease in intensity as a result of its low concentration within the mixture. For GTE, the physical mixture, the 6° peak can be slightly observed in the diffractogram. As these crystalline peaks are present only in the mixtures but not in the nanostructures, it can be concluded that encapsulation was successful.

#### Fourier transform infrared (FTIR) analyses

**Fig. 4:**
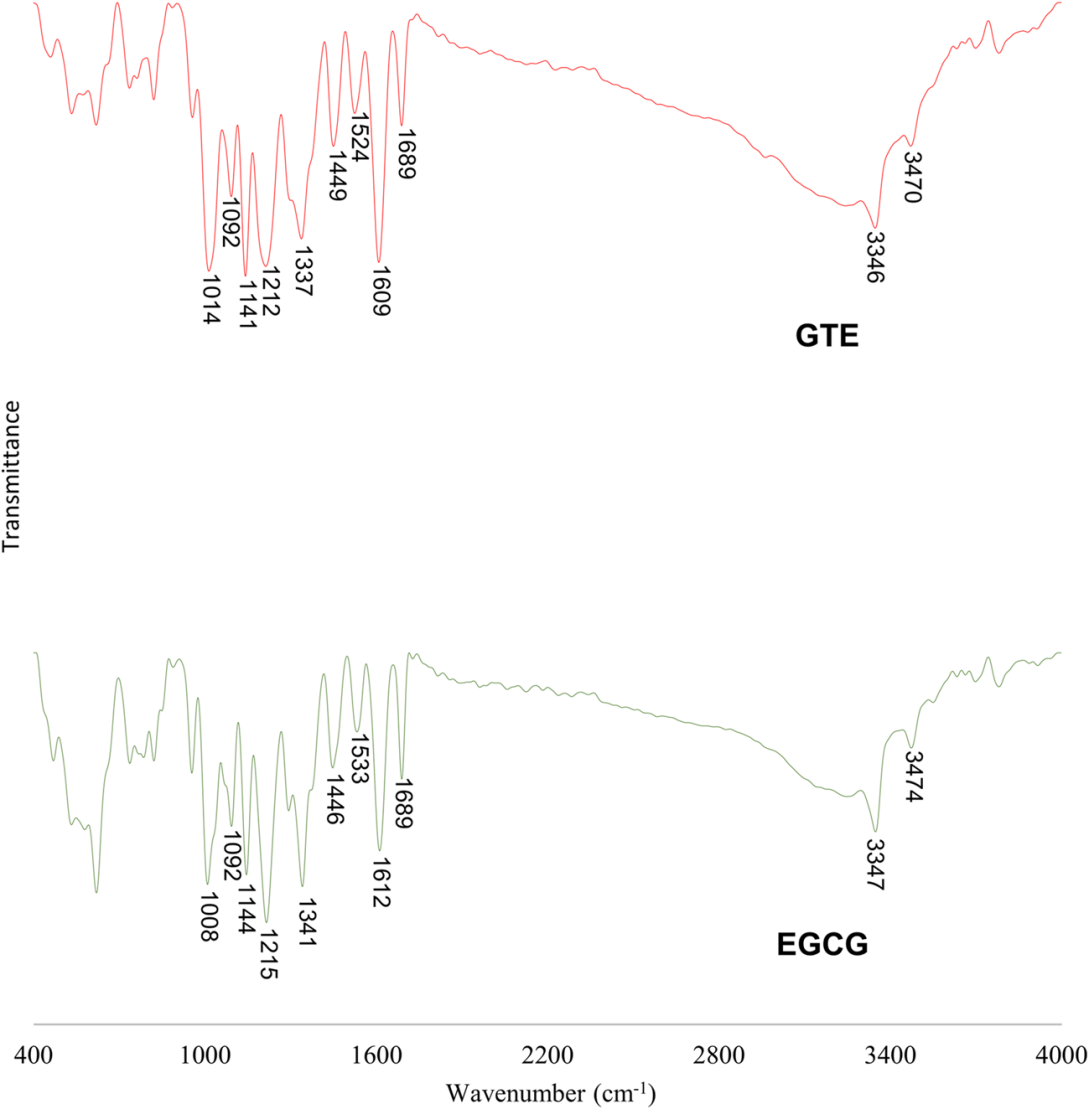
FTIR spectrograms of (**A**) EGCG and (**B**) GTE

Figure 4 displays the FTIR spectrum of pure EGCG and GTE. The EGCG spectra show characteristic peaks at 3474 and 3347 cm^−1^ (O-H stretching), 1689 cm^−1^ (C=O stretching), 1612, 1533, and 1446 cm^−1^ (C=C, aromatic stretching), 1341 cm^−1^ (O-H bending), 1215 cm^−1^ (C-O stretching) and 1144 cm^−1^ (C-O stretching from tetrahydropyran ring), 1092 and 1008 cm^−1^ (aromatic ring stretching) (Billes et al. 2007; Robb et al. 2007; Wang et al. 2019). The GTE spectra were similar to those of EGCG, with slight shifts on some peaks as a result of the presence of other components in lower concentration, indicating that a high concentration of EGCG was found in this commercial extract.

**Fig. 5:**
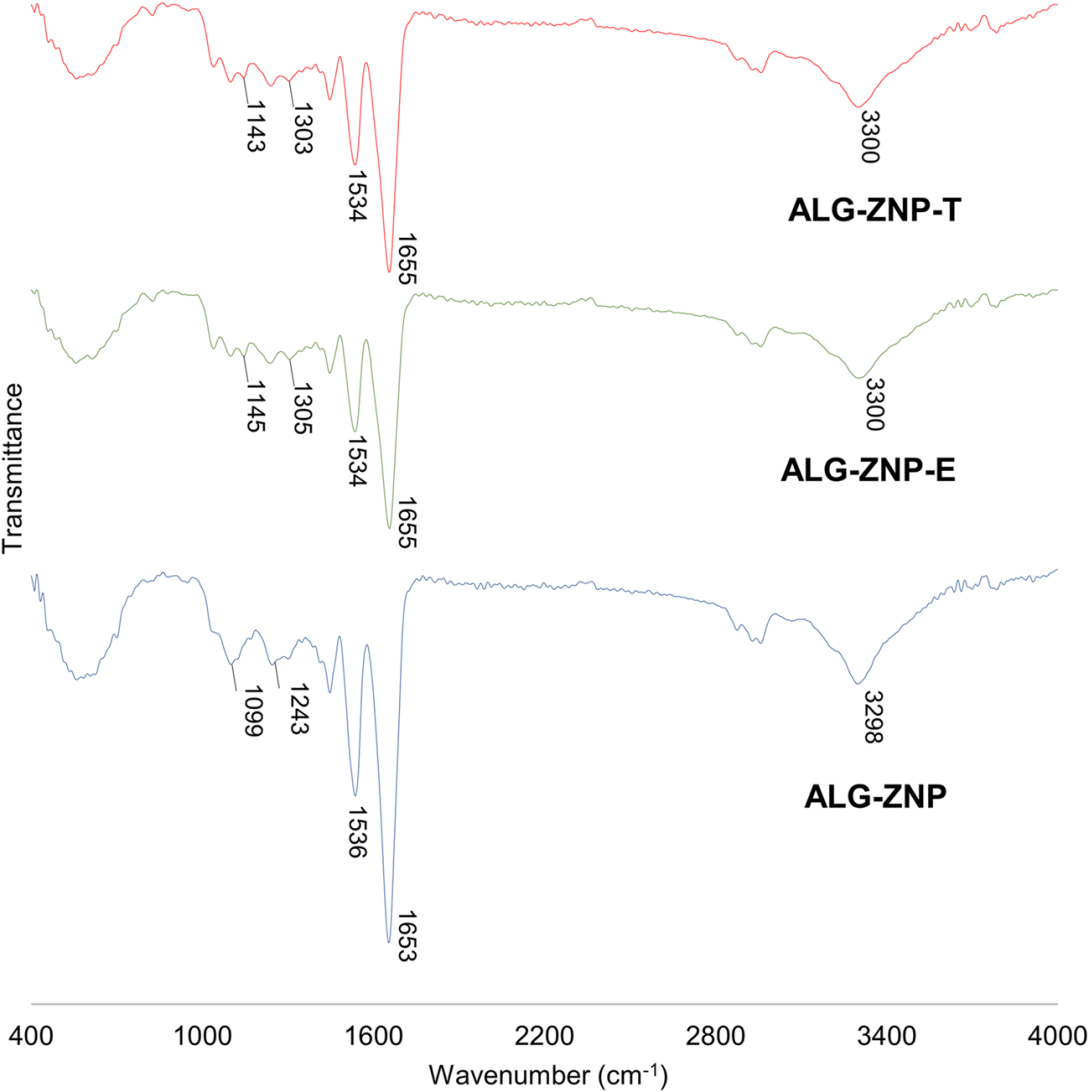
FTIR spectrograms of ALG-ZNP blank nanoparticles and ALG-ZNP-E and ALG-ZNP-T nanoparticles

Figure 5 shows FTIR spectra of ALG-ZNP, ALG-ZNP-E, and ALG-ZNP-T. All nanoparticle formulations had two characteristic peaks of zein at 1653 and 1536 cm^1^, from amide I (C=O stretching) and amide II (N-H bending), respectively (Jin et al. 2022a). ALG-ZNP-E and ALG-ZNP-T displayed a peak around 1144 cm^−1^ derived from the C-O stretching of tetrahydropyran ring. The rest of the EGCG or GTE peaks did not appear, while in the physical mixture of EGCG and GTE with the components of the nanoparticle matrix (Figure S2), polyphenol peaks could be observed. This information suggests that compounds could be found encapsulated in the hydrophobic core of nanoparticles (Khan et al. 2019). Figure S3 displays the FTIR spectra of ALG/CS-ZNP, ALG/CS-ZNP-E, and ALG/CS-ZNP-T; no significant changes resulting from the addition of the chitosan coating were observed in the infrared spectrum.

### Antioxidant activity

As mentioned above, catechins have a great number of biological activities that are beneficial to aquatic organisms. Most of these beneficial biological activities stem from their high antioxidant power. Catechins can exhibit antioxidant power directly, by scavenging free radicals, or indirectly, through the activation of antioxidant enzymes, EGCG being the one with the highest antioxidant power (Bernatoniene and Kopustinskiene 2018). However, its absorption is limited *in vivo* due to its labile nature (Kim et al. 2014), for which we propose its encapsulated use.

**Fig. 6:**
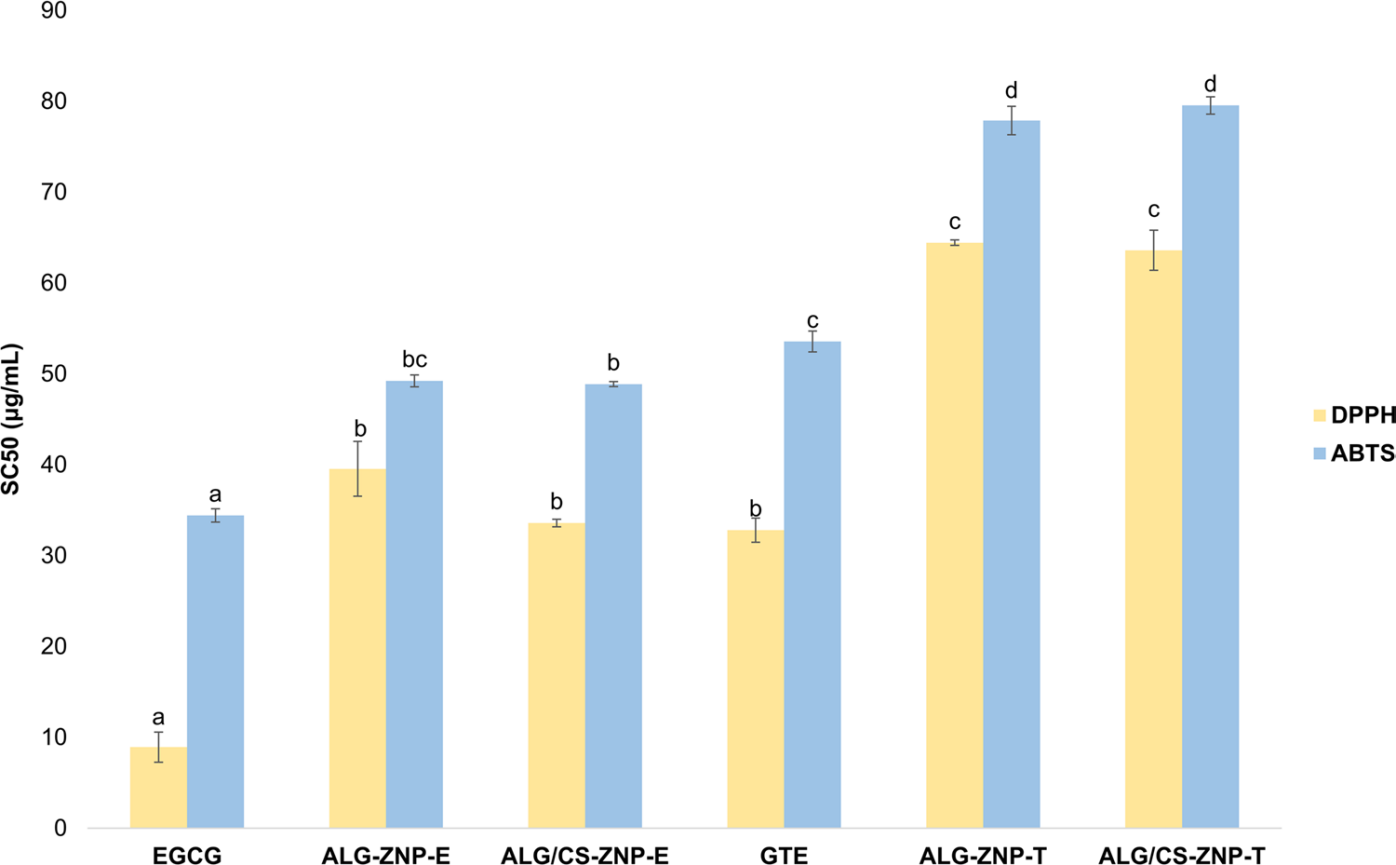
Antioxidant assays (DPPH and ABTS) tested on nanoparticles and free EGCG and GTE. Different letters referring to the same antioxidant compound indicate the existence of significant differences in activity shown by the different types of nanoparticles tested (*P*<0.05)

Free radicals are molecular species with an unpaired electron in their outer atomic orbital. These molecules, due to their high instability, can react with different cellular components (DNA, lipids, and proteins) causing cell damage that may lead to organ failure (Lobo et al. 2010). Therefore, it is important to test the free radical scavenging potential of nanoparticles and encapsulated compounds. The antioxidant power *in vitro* of encapsulated EGCG and GTE has been investigated and compared to that of free substances. Figure 6 shows the SC50 of the DPPH and ABTS assays of the different synthesized nanoparticles compared to the free substances.

Similar trends can be observed for the two tested assays. In general, the minimum concentration to scavenge 50% of free radicals (SC50) is higher when the compounds were encapsulated. This increase in SC50 and, therefore, a decrease in antioxidant power could be due to the fact that when substances are encapsulated, they can form hydrogen bonds in addition to other types of interactions with the encapsulation matrix (Li et al. 2009). These interactions reduce the antioxidant capacity of polyphenols (Gulcin 2020).

The SC50 of EGCG was lower than that of GTE. EGCG accounts for 45% of the composition of GTE, while the rest are mainly other catechins that have less antioxidant power. DPPH showed an SC50 for EGCG and GTE of 8.92 and 32.79 μg/mL, respectively. After zein encapsulation and subsequent alginate coating, the SC50 of ALG-ZNP-E and ALG-ZNP-T increased to 39.54 and 64.43 μg/mL. Chitosan coating caused a decrease in SC50 for ALG/CS-ZNP-E (33.58 μg/mL) with respect to ALG-ZNP-E, although it did not show significant differences. For ALG/CS-ZNP-T, the coating did not significantly affect SC50 (63.59 μg/mL).

In the ABTS radical assay for EGCG and GTE, SC50 increased compared to the DPPH assay, 34.42 and 53.56 μg/mL respectively. However, the response profile obtained was similar to that described previously. Thus, SC50 increased for the encapsulated compounds compared to the free substances. ALG-ZNP-E and ALG-ZNP-T needed 49.23 and 77.86 μg/mL to scavenge 50% of the ABTS radicals. The addition of chitosan did not cause a worsening of the antioxidant power of the nanoparticles. ALG/CS-ZNP-E and ALG/CS-ZNP-T showed an SC50 of 48.87 and 79.54 μg/mL. According to Osman et al. (2006), the oxidation process experienced by polyphenolic compounds takes place at different positions depending on the method used, which could explain the differences observed in the results obtained for each of the protocols applied. Additionally, this could imply that the interactions between the nanoparticle constituents and the active substances they encapsulate occur at specific positions. The decrease in the availability of these specific positions would therefore affect the antioxidant activity assay differently depending on the substrate used.

These results show that the antioxidant power of catechins present in nanoparticles is diminished by encapsulation. However, considering that encapsulation confers protection to active materials against hostile conditions they would face in their use in the aquaculture sector, such as those inherent in digestive processes, the antioxidant power still retained by nanoparticles can be considered adequate (Khan et al. 2019; Liang et al. 2021; Pauluk et al. 2019).

### Antimicrobial activity assay

**Fig. 7:**
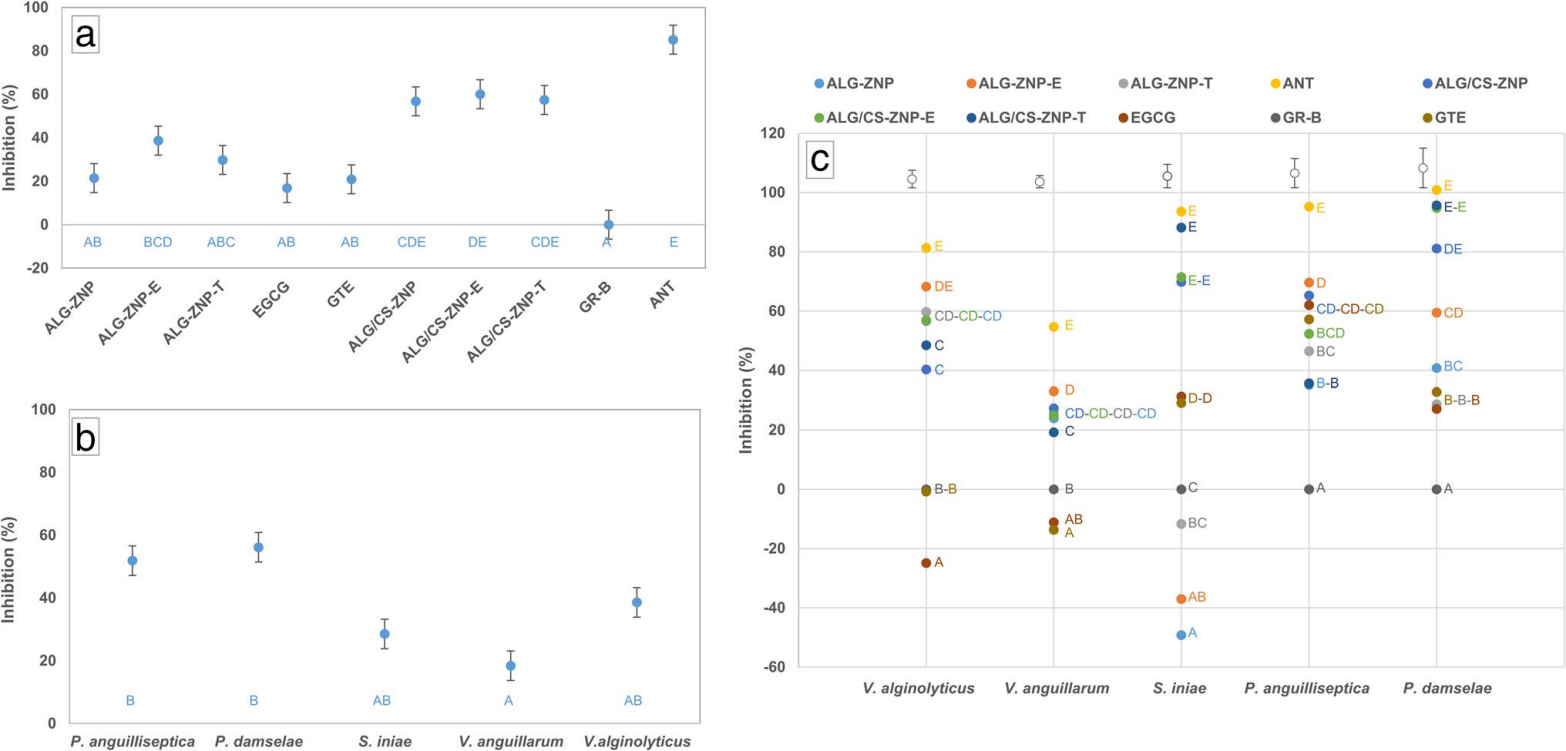
Antimicrobial activity associated with the different formats of active substances and nanoparticles tested. **a** Overall activity as a function of the type of substance. **b** Global activity as a function of the target microorganism. **c** Individualized activity for each of the bacteria tested. In all cases, the mean values and the corresponding standard error are shown. The letters associated with each value reflect the homogeneity groups generated by the Tukey HSD test, with global character in cases A and B, and associated with bacterial species in case C. ALG-ZNP: alginate NP; ALG-ZNP-E: alginate NP with EGCG; ALG-ZNP-T: alginate NP with green tea extract; GTE: green tea extract; ALG/CS-ZNP: alginate/chitosan NP; ALG/CS-ZNP-E: alginate/chitosan NP with EGCG; ALG/CS-ZNP-T: alginate/chitosan NP with green tea extract; GR-B: growth control; ANT: antibiotic

Figure 7a shows the results obtained in relation to the antimicrobial activity shown by the different active ingredients tested and the different formats in which they were tested.

Considering the results from a global perspective and with the type of material tested as a unifying criterion, the antimicrobial activity exhibited by the nanoparticles with the presence of chitosan stood out, the only ones that, with inhibition levels between 56 and 60%, were close to the values reached by the antibiotic (85%), established as a positive control. The rest of the formats tested did not exceed 40% inhibition in any case, although the nanoparticles with alginate and encapsulated product, both EGCG and tea extract, did not generate significant differences, for the most part, with the chitosan formats mentioned above. The lowest inhibitory capacity was observed for alginate nanoparticles without active product, as well as for the active products themselves in free format, with percentages ranging from 16 to 21%. Encapsulation of materials with antimicrobial activity in mixed alginate-chitosan nanoparticles has been shown to be a suitable practice to increase the efficiency of such materials (Yoncheva et al. 2021; Zimet et al. 2018). However, and based on the results obtained in the present study, with the majority absence of significant differences between the inhibitory capacity of empty nanoparticles and that of nanoparticles with active materials, as reported in other works (Zaidan and Kadhum 2020), it is worth asking whether the activity of such materials is actually increased. In the case of alginate nanoparticles, such an effect seems to be present, especially in the case of EGCG, the only case in which there was a significant increase in the percentage of growth inhibition, both with respect to empty nanocapsules and free EGCG. In contrast, in the case of nanoparticles with the additional presence of chitosan, although the inhibitory capacity was higher in those that included EGCG, the increase was not sufficiently intense to generate a statistical difference. In this case, it would be attributable, therefore, mostly to the components of the nanoparticles and, in particular, to the chitosan. Friedman et al. (2013) also observed significant inhibitory activity of empty nanoparticles, attributed to the affectation of the lipid fraction of the cytoplasmic membrane. Furthermore, and similar to what was described in the present work, chitosan was shown to be mainly responsible for the potential to reduce microbial growth. It follows, under these conditions, that not all amino groups present in chitosan, which confer the polymer with a positive charge, are counteracted by the negative charge of carboxylic acids associated with alginate, leaving the potential to interact with the cell cytoplasmic membrane through their negative charges (Al-Gethami and Al-Qasmi 2021). However, it is not advisable to generalize these conclusions, since the results associated with each of the components can vary depending on the microorganism used in the assay (Asadpoor et al. 2021; Paiva Filho et al. 2020).

The difference in response offered by different microorganisms can be seen in Fig. 7b. This figure shows *Vibrio anguillarum* as the species least overall sensitive to the action of the different formats tested, with an average percentage of growth inhibition slightly higher than 18%, while *Photobacterium damselae* was the most affected (56.12%), although with little difference with respect to *Pseudomonas anguilliseptica* (51.89%). Between the two extremes were *Streptococcus iniae* and *Vibrio alginolyticus*, statistically related to the two previous groups. The existence of different responses to the same compound is common not only between different species (Ignasimuthu et al. 2019) but also between strains of the same species (Siriphap et al. 2022). The mechanisms through which EGCG and other green tea polyphenols act are diverse (Reygaert 2018), as are the pathogenic strategies adopted by different strains and the specific composition of their cell structures; therefore, varied responses are expected in assays of this nature. Generally speaking, Gram-positive species are considered to be more sensitive to polyphenols (Zhang et al. 2014). With regard to EGCG, and in nanoparticle format, a higher resistance of Gram-negative bacteria is postulated as a consequence of the existence of the outer membrane and lipopolysaccharide, which limits the potential of nanoparticles to bind to the peptidoglycan layer (Zhao et al. 2022b). The sensitivity exhibited by certain Gram-negative species would be more related to the production of oxidative damage (Cui et al. 2012). In the case of *V. alginolyticus*, the ability of phenolic compounds to reduce their biofilm formation capacity has been demonstrated, among other factors, by affecting the biosynthetic potential of polysaccharides, in addition to altering the permeability of the cytoplasmic membrane (Liu et al. 2021). In the case of *P. damselae*, bacteria that generally register higher levels of sensitivity to polyphenolic compounds than other fish pathogens (Bulfon et al. 2014), as observed in the present study, the possible impairment of biofilm-forming capacity as well as motility potential has also been postulated (Bautista-Rosales et al. 2022). In the case of *V. anguillarum*, the species for which the lowest degree of inhibition was recorded, compounds of a different nature, such as antimicrobial peptides or polyunsaturated fatty acids, appear to be more effective in their control (Citarasu 2012).

Figure 7c shows the individual response of each microorganism for each of the formats tested. The levels of growth inhibition obtained in each case confirm what was previously mentioned with respect to the variability detected according to the microorganism studied. Thus, *S. iniae* and *P. damselae* showed differentially significant sensitivity between nanocapsules with and without chitosan incorporation. In both cases, the levels of growth inhibition experienced by both bacteria were clearly higher for the former, and the Gram-positive species even showed a higher degree of cell development in the medium with nanocapsules without chitosan than in the control free of antimicrobial compounds. The greater efficacy of nanoparticles with chitosan in this case may be due to the interactions between the positive charges provided by this polymer and the negative charges of the teichoic acids present in the Gram-positive cell wall (Kaur et al. 2020), thus favoring the altering potential on the cytoplasmic membrane and, therefore, its antimicrobial capacity (Raafat et al. 2008). Regarding *P. damselae*, and although most studies point to a lower sensitivity of Gram-negative species to chitosan (Li and Zhuang 2020), this polymer has also shown interesting levels of inhibitory activity in relation to certain bacteria of this group. In the most sensitive species, it is postulated that its greater degree of affectation could be related to a higher hydrophilic character, which would favor the access of the compound to the cellular interior in greater proportion (Chang et al. 2004). On the contrary, *V. alginolyticus* and *V. anguillarum* were affected to a greater extent by nanoparticles consisting only of alginate, especially those carrying EGCG. In this case, the inhibition levels achieved led to significant differences with all of the chitosan nanoparticles in the case of the first bacterium, while in the case of the second, the significance was limited to the chitosan nanocapsules with green tea extract. The last of the tested species, *P. anguilliseptica*, showed a less defined pattern, with inhibition values close for all formats, which means that few of them differed significantly from each other. As previously discussed, chitosan nanoparticles seem to show lower efficiency on Gram-negative species, although the results reflected in the existing literature are somewhat contradictory (Chandrasekaran et al. 2020). The great variability that exists in terms of the conditions under which the studies are carried out, especially with regard to the presence of additional materials in the nanoparticles, makes it difficult to obtain homogeneous results and therefore unique conclusions. However, and depending on the mechanisms of action mostly recognized for chitosan (interaction with different negatively charged structures present in cell envelopes), the specific molecular architecture associated with each species can determine and condition different degrees of sensitivity to chitosan nanoparticles (Duan et al. 2019). Regarding the active substances tested, EGCG and green tea extract, a similar pattern was observed for all bacteria, so no statistically significant levels of inhibition were found between nanocapsules of the same type that carry different antimicrobial substances. This last result is quite positive from an economic perspective, given the great difference in cost between a natural extract and a pure compound from this extract and the importance that this factor reaches in industrial processes.

These nanoparticles especially ZNP coated by ALG and CS can be a suitable delivery system for tea polyphenols and other biologically active substances. Other studies found that a similar encapsulation format to those used in this experiment improves the bioaccessibility of encapsulated compounds (Carrasco-Sandoval et al. 2021; Khan et al. 2019) and increases the stability of the encapsulated substances and photostability during storage (Luis et al. 2020; Zhang et al. 2023). This protection is highly relevant for the inclusion of these formulations in aquaculture feeds due to the different storage conditions that the feed may face.

## Conclusions

The encapsulation of EGCG and GTE in ZNP was performed by an antisolvent method, stabilizing the ZNP obtained by a layer-by-layer method with alginate and chitosan. The obtained nanoparticles had a spherical shape, with a maximum size of 260 nm and an encapsulation efficiency of GTE and EGCG greater than 75% in all cases. The results found that these formulations were able to maintain a large part of their antioxidant activity with respect to free substances. In relation to antimicrobial activity, the growth inhibition potential showed a dependence on the pathogenic species under study. The presence of chitosan coating on the nanoparticles increased the percentage of growth inhibition, reaching approximately 60% inhibition on average for all pathogenic species, and even for *P. damselae*, the presence of chitosan coating produced levels of growth inhibition close to the antibiotic, used as a positive control. These results open new paths for the use of these nanoparticles in the control of diseases and/or possible synergy with the antibiotic, thus reducing the amount needed. In addition, encapsulation protects the substance against degradation and can be stored as an isolated product or as part of the formulation in aquaculture diets. Further studies are needed to examine the release of substances in the digestive process, as well as their stability in dispersion or as dry material.

### **Supplementary information**

ESM 1 (DOCX 1101 kb)
