# Supplementary material for: Antimicrobial and antioxidant activity of encapsulated tea polyphenols in chitosan/alginate-coated zein nanoparticles: a possible supplement against fish pathogens in aquaculture
Source: Environ Sci Pollut Res Int. 2024 Jan 23;31(9):13673–87. doi: 10.1007/s11356-024-32058-x (PMC10881692; doi:10.1007/s11356-024-32058-x)
Supplement: Supplementary file 1 — (DOCX 1101 kb) [file 11356_2024_32058_MOESM1_ESM.docx]

**Supplementary material**


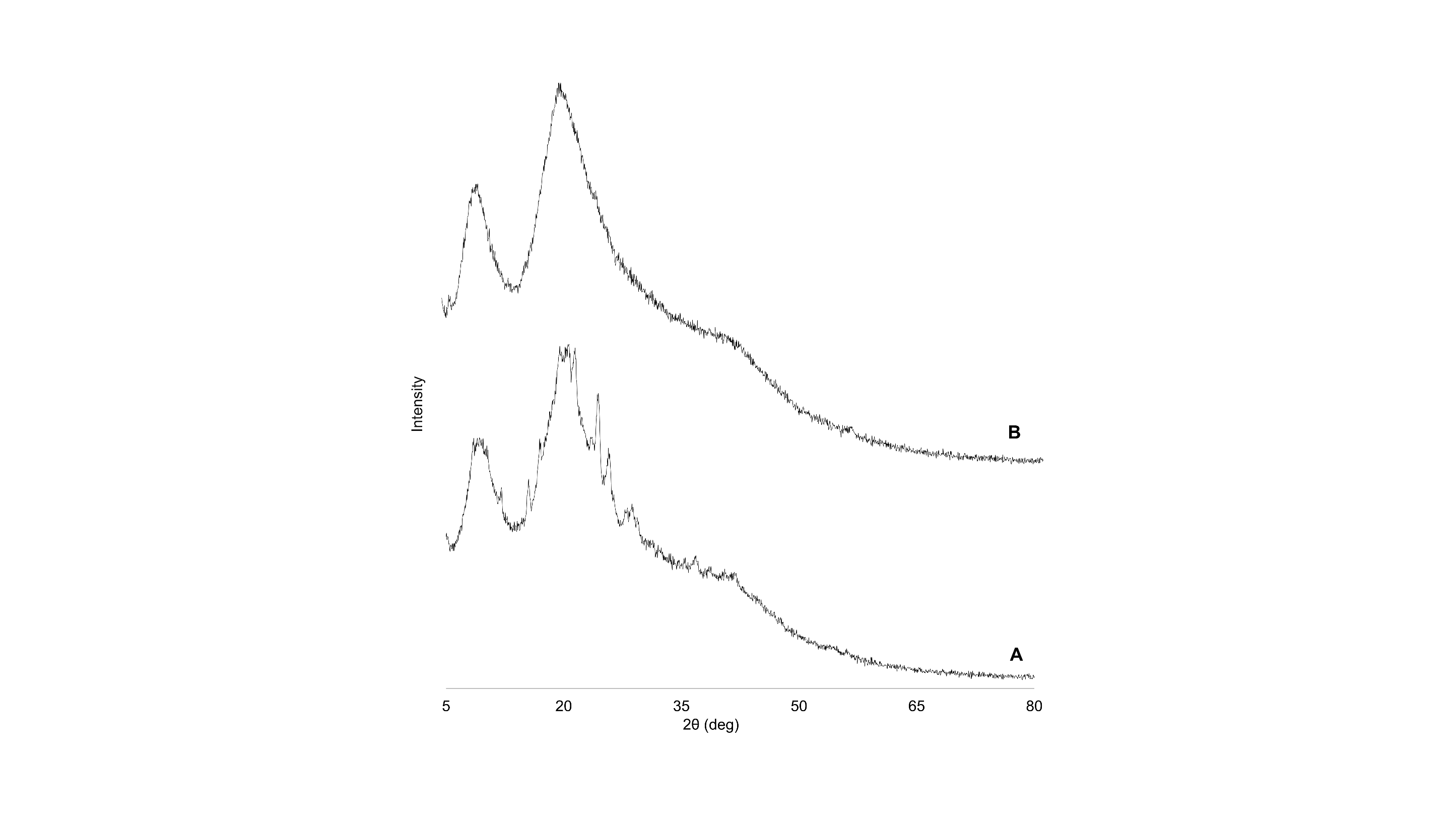


**S1**. XRD diffractogram of physical mixture of EGCG, alginate, chitosan and zein (A) and GTE, alginate, chitosan and zein (B).


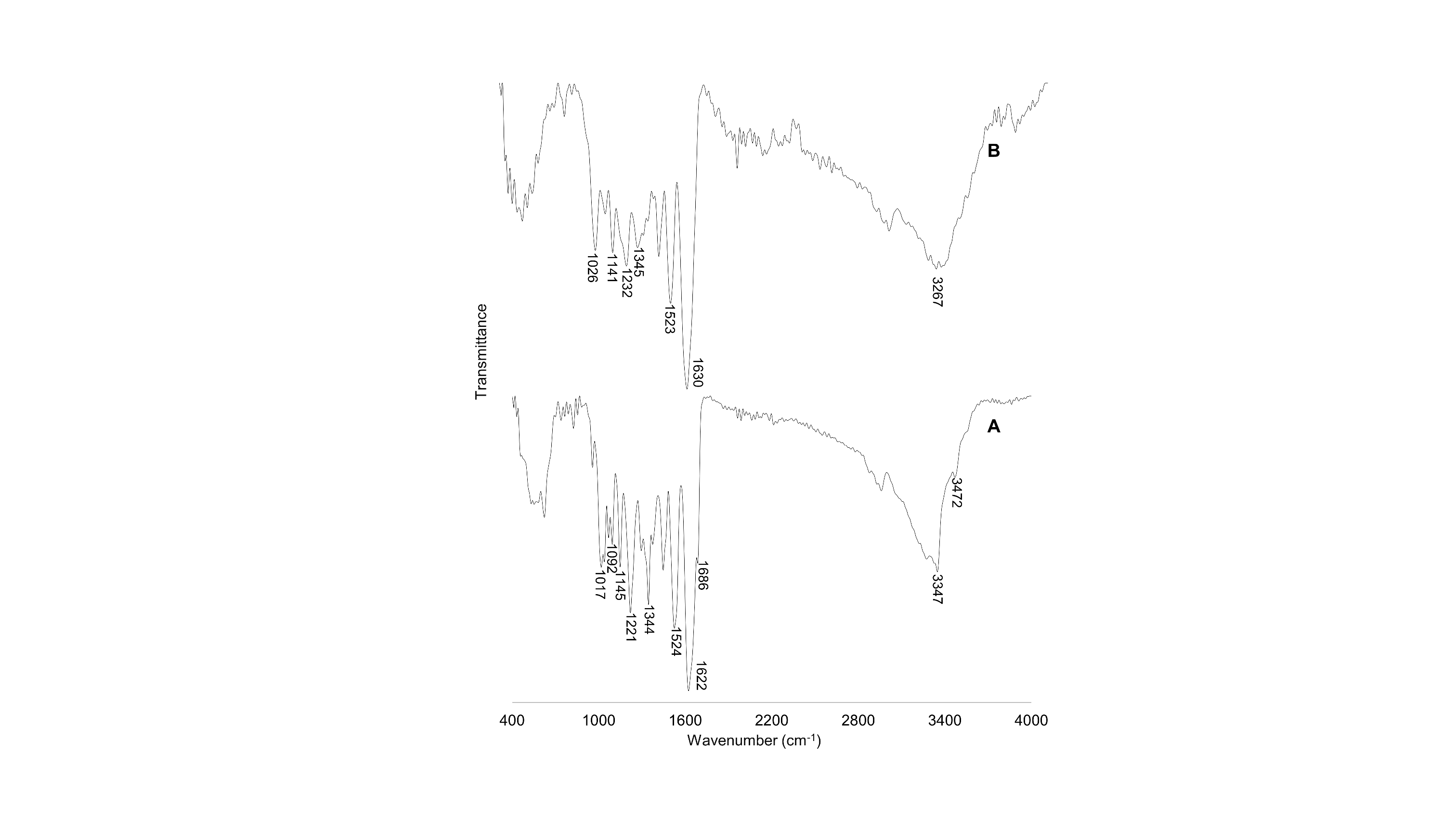


**S2**. FTIR spectrum of physical mixture of EGCG, alginate, chitosan and zein (A) and GTE, alginate, chitosan and zein (B).


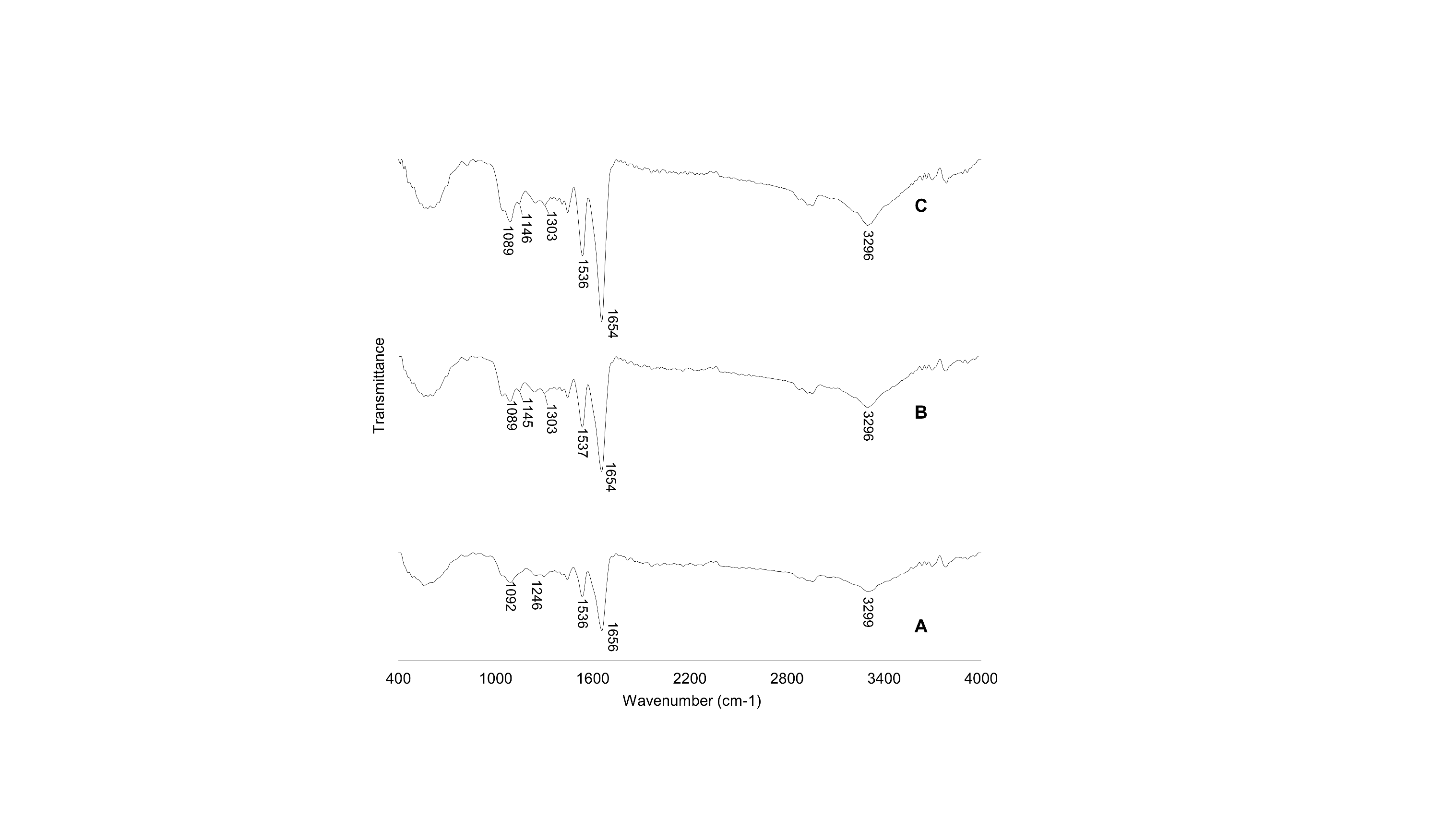


**S3**. FTIR spectrum of ALG/CS-ZNP (A), ALG/CS-ZNP-E (B) and ALG/CS-ZNP-T (C)
